# Development of a Drug-Loaded Shape-Memory Polymer Urethral Stent (SMPUS) for Treatment of Prostatic Urethral Obstruction (PUO)

**DOI:** 10.1021/acsomega.5c05903

**Published:** 2025-08-07

**Authors:** Alaa Fehaid, Koichiro Uto, Toshimasa Homma, Ailifeire Fulati, Maëlys Tisserant, Jun Nakanishi, Mitsuhiro Ebara

**Affiliations:** † Research Center for Macromolecules and Biomaterials, 52747 National Institute for Materials Science (NIMS), 1-1 Namiki, Tsukuba, Ibaraki 305-0044, Japan; ‡ Division of Chemical Engineering and Biotechnology, National Institute of Technology, Ichinoseki College, Takanashi, Hagisho, Ichinoseki, Iwate 021-8511, Japan; § Forensic Medicine and Toxicology Department, Faculty of Veterinary Medicine, Mansoura University, Mansoura, Dakahlia 35516, Egypt; ∥ Department of Advanced Materials Science, Graduate School of Frontier Sciences, The University of Tokyo, Chiba, 277-8561, Japan; ⊥ 300657 Graduate School of Pure and Applied Sciences, University of Tsukuba, 1-1-1 Tennodai, Tsukuba, Ibaraki 305-8577, Japan; # Graduate School of Advanced Engineering, Tokyo University of Science, 6-3-1 Katsushika-ku, Shinjuku, Tokyo 125-8585, Japan

## Abstract

Prostatic urethral obstruction (PUO) is the urethral narrowing by the external pressure of an enlarged prostate, which hinders the ability to pass urine normally. The most common reasons for an enlarged prostate are prostate cancer and benign prostate hyperplasia. In this study, we aimed to develop a drug-loaded shape-memory polymer urethral stent (SMPUS) to address PUO. The SMPUS was designed to combine both mechanical and chemical/herbal treatment of PUO. Successfully, SMPUS shows potential mechanical efficacy through its shape-memory properties that ensure the patency of the urethral lumen by restoring its tube-like stent shape upon flushing with hot water at its melting temperature. Additionally, the in vitro drug release investigation shows the capability of SMPUS to serve as a versatile drug delivery platform, after further in vivo investigation, enabling the release of the selected drugs to treat the primary diseases associated with PUO while mitigating the systemic side effects associated with conventional treatments. Both polymer and drug concentrations can be modulated to achieve the required mechanical and thermal properties based on the case progress. This adaptability of the developed SMPUS allows for future in vivo and clinical trials in medical applications.

## Graphical abstract

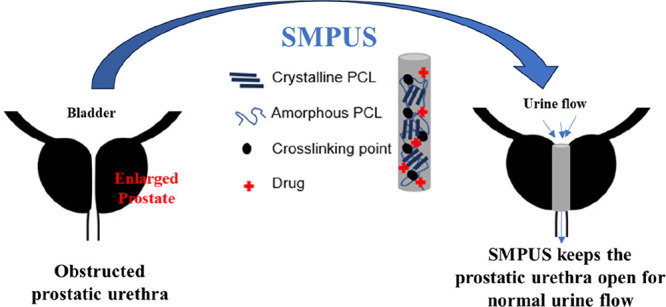

## 1 Introduction

The prostate is a small gland in the male genitourinary system that surrounds the urethra just after the urinary bladder neck. Its main function is to produce seminal fluid and regulate hormone production and urine flow.^1^ Prostatic cancer (PCa) and benign prostatic hyperplasia (BPH) are the major diseases of the prostate. PCa is considered the fifth leading cause of death from cancer, with 6.6% of the total male cancer mortalities.^2^ BPH cases are increasing at an alarming rate worldwide, and it is expected to increase more in the coming years.^3^ PCa and BPH share some similarities, including the fate of the prostatic urethral obstruction (PUO) because of the enlarged prostate.^4^ PUO is the blockage (partially or totally) of urine flow through the urethra because of urethral narrowing by outer pressure from the prostate, which hinders the ability to pass urine normally. The severity of lower urinary tract symptoms (LUTS) differs according to the degree of both the urethral obstruction and prostatic enlargement; symptoms usually include difficulties initiating urination, pain, frequent urination, and, sometimes, blood in the urine.^5,6^

The most common approach for PUO treatment is surgery using transurethral resection of the prostate (TURP) as the gold standard method. However, many elderly men are nonresponders to TURP, either because they are unsuitable for the required anesthesia or because of the very large size of their prostate, and need an alternative way of treatment.^7–9^ Prostatic stents as an alternative treatment for PUO were first used by Fabian in 1980 and have gone through different developments later.^10^ The principle of the prostatic stent is to insert a tube-like supporting instrument into the urethra to keep the hollow lumen of the prostatic urethra and restore the normal urine flow.^11^ The optimal criteria for prostatic stents include ease of insertion without the need for general anesthesia, improvement in LUTS, maintenance of continence, resistance to encrustation, and avoidance of urinary tract infections.^12^ Many types of stents have been developed during the past few years such as permanent stents that are mainly metal stents embedded in the urethra; however, in 40% of the patients, the stents were removed because of stent migration, encrustation, and excessive cell proliferation.^13^ Different generations of temporary expandable stents were then developed and reported for their easier insertion and lower incidence of urethral injury. Temporary stents are made from rust-resistant stainless steel, biocompatible nitinol, and thermo-expandable nickel–titanium alloy materials with different modifications, but because of the metal encrustation and migration rate, their clinical use is still limited and still needs a removal interference.^14–16^ To avoid metal encrustation, stents have been developed using polymer-based materials and were successfully reported to allow easy insertion initially; however, many patients later experienced urinary retention and irritation.^17^ Using biodegradable stents is promising nowadays; polylactic acid (PLA) and polyglycolic acid stents have been used to solve migration and fragmentation issues. However, the mechanical efficiencies of these stents should be improved and tested clinically.^18,19^

The currently developed stents can provide only a mechanical way of therapy for the PUO. Therefore, chemical therapy is always prescribed together with the mechanical stents such as anticancer chemotherapy (Docetaxel, Doxorubicin, and many others), 5-alpha reductase inhibitors (Finasteride), or alpha-blockers (Doxazosin) in the case of BPH.^20,21^ Drugs can relieve LUTS, but since they are systematically administrated, they induce different side effects (hair loss, digestive disorders, fluid retention, and respiratory troubles) and cannot cure the disease permanently.^20,21^ Therefore, in this study, we aimed to design a biodegradable drug-loaded shape-memory polymer urethral stent (SMPUS) that combines both mechanical and chemical or herbal therapies.

The technology of synthesis of smart biodegradable shape-memory polymer (SMP)—chemically cross-linked poly­(ε-caprolactone) (PCL)—has been successfully established in our laboratory.^22,23^ The PCL used in Food and Drug Administration (FDA)-approved devices is a biodegradable synthetic material with high biocompatibility and no toxicity.^24^ The shape-memory properties allow it to recover its permanent original shape—after fixation in a temporary deformed shape—in response to external stimuli such as heat and light.^23,25^ SMP exhibits different dynamic motions such as twisting, bending, lifting, and stretching, which makes it one of the most feasible materials in biomedical applications.^26,27^ Different SMP-based medical devices have been developed recently such as the shape-memory balloon that offers thermal and chemical therapies for osteosarcoma.^28^ A shape-memory vascular stent was also developed for arterial stenosis treatment.^29^ Moreover, an SMP-based string could be developed to contract blood vessels in fetal surgeries.^30^ That is why using the SMP to develop a prostatic urethral stent was considered promising as their folded state allows for easy insertion, which is one of the most important criteria for stents.

Moreover, both chemical (Doxorubicin) and herbal (Aescin) drugs were chosen to be loaded into the SMPUS in this study to investigate the possibilities of drug release. PCL was used widely to fabricate nanofibers by electrospinning, and many drugs (Doxorubicin, Paclitaxel, Temozolomide) were successfully incorporated into those nanofibers, and then their release behaviors were reported,^28,31,32^ suggesting the PCL as a promising material for drug delivery systems.

In this study, we successfully fabricated for the first time a drug-loaded SMPUS that can be easily inserted into the prostatic urethra through the external urethral orifice using its folded state as a temporary deformation and also can keep the lumen of the prostatic urethra open after recovering the permanent tube-like shape in response to heat as an external stimulus, along with localized drug release.

## 2 Materials and Methods

### 2.1 Chemicals

Pentaerythritol, ϵ-caprolactone, tin­(II) 2-ethyl hexanoate, benzoyl peroxide (BPO), and doxorubicin hydrochloride (Dox) were purchased from Tokyo Chemical Industry (TCI. Co., Ltd. Japan). Triethylamine, tetrahydrofuran, acryloyl chloride, hexane, super dehydrated *N*,*N*-dimethylformamide (DMF), acetone, and methanol were purchased from Wako Pure Chemical Industries Ltd., Japan. Aescin was purchased from Sigma-Aldrich (Merck, Darmstadt, Germany).

### 2.2 SMP Synthesis

4b50 PCL was synthesized by the bulk polymerization method as follows. 644 mg of the initiator (tetravalent alcoholic compound, pentaerythritol) was placed in a dry round-bottom flask and kept under reduced pressure overnight for complete drying. Then, 100 mL of the monomer (ϵ-caprolactone) was added by a glass syringe under a flowing nitrogen atmosphere and kept at 80 °C in an oil bath for 30 min to allow the dissolving of the initiator in the monomer. Then, 0.5 mL of the catalyst (tin­(II) 2-ethyl hexanoate) was added slowly, and the reaction was carried out overnight at 120 °C under a nitrogen atmosphere. After that, the solution was diluted using 500 mL of tetrahydrofuran and reprecipitated with 2500 mL of hexane overnight at 4 °C. The residual solvent was completely removed, and the precipitate was well-dried under reduced pressure of the vacuum pump overnight. To get a highly purified product, it was reprecipitated with hexane 3 times.

Then, the 4b50 PCL macromonomer was synthesized by dissolving 100 g of 4b50 PCL in 400 mL of dehydrated tetrahydrofuran using a magnetic stirrer for 2 h. After dissolving, 15 mL of dried triethylamine and 7.2 mL of acryloyl chloride were added slowly in an ice bath for 10 min and then wrapped with aluminum foil and kept overnight at 4 °C. The solution was then reprecipitated with 2500 mL of cold methanol. After the precipitation process was repeated and the purified 4b50 PCL macromonomer was obtained, it was dried under reduced pressure overnight. Proton nuclear magnetic resonance spectroscopy (1H NMR spectra, JEOL, Tokyo, Japan) was used to characterize the chemical structure of 4b50PCL.

### 2.3 Fabrication of Drug-Loaded SMPUS

SMPUSs were fabricated via polymerization of the thermal initiator BPO and the 4b50PCL-macro. 4b50PCL-macro was dissolved in DMF with corresponding polymer concentrations (30, 40, 50%), and different drugs and BPO were added with corresponding concentrations. The mixture solution was cast into a Teflon tube-like mold with dimensions of 10 mm diameter, 50 mm height, and 0.5 mm thickness. The cross-linking of the solution was performed at 80 °C for 5 h. After that, the cross-linked SMPUSs were immersed in acetone for a few seconds to remove the un-cross-linked molecules and then rinsed with methanol. Finally, the SMPUSs were dried by a vacuum pump under reduced pressure overnight.

### 2.4 Characterizations of Mechanical Properties

To characterize the mechanical properties of the fabricated SMPUSs, films using the same prepared mixtures were fabricated by sandwiching between two glass slides with a 0.5 mm thick Teflon spacer between them (the same thickness as SMPUSs). These films were characterized by using a tensile tester (EZ-S 500N, Shimadzu, Kyoto, Japan). All measurements were adjusted for an elongation rate of 5 mm min^–1^ at room temperature. The stress–strain curves were obtained from the recorded force and stroke data of the tensile testing for three different batches of each film using the following equations:

$$\mathrm{Stress}\;(\mathrm{MPa})=\mathrm{Force}\;(N)÷\mathrm{Surface}\;\mathrm{area}\;({\mathrm{mm}}^{2})$$

Strain(%)=(Stroke÷Gaugelength)×100

### 2.5 Characterizations of Thermal Properties

Differential scanning calorimetry (DSC 600, thermal analysis system, Hitachi High-Tech Science, Japan) was used to characterize the thermal properties of the fabricated SMPUSs. Samples were measured in a temperature range from 0 to 100 °C, at an increase rate of 5 °C min^–1^. The degree of crystallinity (χ_c_%) was calculated by using the following equation:

$$\chi_{c}(\%)=(\Delta H÷\Delta H_{m})\times 100$$

Δ*H* represents the melting enthalpy for each SMPUS, and Δ*H*_m_ represents the melting enthalpy for 100% crystalline PCL, which is reported to be 136 J g^–1^.^33^

### 2.6 Drug Release Assay

The release behavior of drugs from the fabricated SMPUSs was investigated in vitro. SMPUS samples were immersed in a vial containing a certain volume of phosphate-buffered saline (PBS) for 60 days at 37 °C and slightly shaken using a magnetic stirrer. At predetermined points of time, a portion of PBS of different samples was withdrawn from the bath and replaced with fresh PBS depending on the withdrawn volume of PBS sample. After that, the Aescin absorbance in the withdrawn PBS sample was measured at 251 nm using a spectrophotometer (V-770 spectrophotometer, Jasco, Tokyo, Japan). Doxorubicin fluorescence signals were measured at a wavelength of 480 nm for excitation and 490 nm for emission using an Infinite 200 pro plate reader (Tecan Trading AG, Switzerland). The concentrations were calculated based on the created standard calibration curves of gradient concentrations of both Aescin and Dox dissolved in PBS and PBS/DMF, respectively. All data are presented as the mean ± SD with *n* = 3. The used SMPUS samples as well as the bath volume in the drug release assay were different between each batch, resulting in different initial concentrations of the drugs that were loaded into each SMPUS sample. Therefore, the percentage (%) was used to express the drug release results as being representative of all samples. The initial loaded amounts of the drug were between 50 and 250 mg. The used equation for the calculation of the released drug percentage is as follows:^28,34^

$$W_{n}\;(\mu g)=C_{n}\;(\mu g\;{\mathrm{mL}}^{-1})\times \mathrm{bath}\;\mathrm{volume}\;(\mathrm{mL})\times \mathrm{dilution}\;\mathrm{factor}$$

$$\mathrm{Release}\;\mathrm{amount}\;(\mu g)=W_{n}-\left(W_{n-1}\times \frac{\mathrm{bath}\;\mathrm{volume}-\mathrm{sample}\;\mathrm{volume}\;(\mathrm{mL})}{\mathrm{bath}\;\mathrm{volume}\;(\mathrm{mL})}\right)$$

$$\mathrm{Cumulative}\;\mathrm{release}\;\mathrm{percentage}\;(\%)=\frac{\sum \mathrm{release}\;\mathrm{amount}\;(\mu g)}{\mathrm{Initial}\;\mathrm{loaded}\;\mathrm{amount}\;(\mu g)}\times 100$$

Here, *W*_*n*_ is the amount of drug in the bath at predetermined time points (*n*), *C*_*n*_ is the drug concentration in the withdrawn PBS sample at predetermined time points (*n*), and *W*_*n*–1_ is the amount of drug in the bath at the previous point of time (*n*).

### 2.7 In Vitro Mimicking of PUO to Test the SMPUS Efficiency

To mimic the PUO, freshly dissected porcine male genital organs were employed for this purpose. It was purchased as harvested organs from slaughtered male pigs from the Shibaura Zouki slaughtering house, Tokyo, Japan. Rubber bands were used to induce outer pressure in the prostate’s area to obstruct the prostatic urethra. Two different degrees of obstruction (slight and severe) were induced by controlling the outer band’s pressure. Then, the fabricated SMPUSs were stimulated by heating using hot water (50–55 °C) to minimize the diameter to the lowest using the folding motion (manual folding) for easy insertion from the external urethral orifice toward the prostatic urethral level using a thin metal inserting tool in the folded temporary shape. After insertion, hot water was flushed into the urethra using a syringe to stimulate the shape-memory property of the stent to recover its original stent shape (tube-like permanent shape) and keep the prostatic urethral way open after shape fixity by cooling down. The sizes of all of the used SMPUSs in this test were 10 mm in diameter, 30 mm in length, and 0.5 mm in thickness. The urine flow through the urethra was then mimicked using a controlled water pump to allow the water to flow at a relatively constant pressure through both normal and obstructed urethra. The volumes of the water that flowed were recorded within 30 s before and after inserting our fabricated SMPUSs in the obstructed urethras. All tests were performed in triplicate to ensure reproducibility and were video-recorded as well. Data are presented as mean ± SD, *n* = 3.

### 2.8 Statistical Analysis

All data were statistically analyzed using the SBSS software program. The obtained values were presented as the mean + standard deviation (SD). One-way ANOVA with Duncan's multiple comparison tests was used to determine the differences between different groups, if applicable.

## 3 Results

### 3.1 SMP Synthesis and Characterization

The structure of the tetrabranched PCL macromonomer with a branch chain length of 50 (4bPCL50) was confirmed by using ^1^H NMR spectroscopy. The degree of polymerization was calculated to be 51.2, while the conversion rate into the 4bPCL50 final polymer was 96.8%.

The mechanical properties were assessed using the tensile testing of the fabricated shape-memory films with a thickness of 0.5 mm at room temperature. As shown in the stress–strain curves (Figure 1), by applying the tensile stress, the control films of cross-linked 4b50PCL with different weight percents of 30, 40, and 50 wt % exhibited elongations of 275, 380, and 540%, respectively. In the films containing Dox and Aescin with 30 wt % 4b50PCL, the strain percent was reduced to 230 and 200%, respectively. Similarly, 40 and 50 wt % 4b50PCL films with Dox and Aescin exhibited strain at breaking of (360 and 350%) and (435 and 430%), respectively. The drug content in the PCL films did not significantly change the stress–strain curves, indicating that it had no substantial impact on the mechanical properties of the PCL films and the fabricated SMPUSs in the current application.

**Figure 1:**
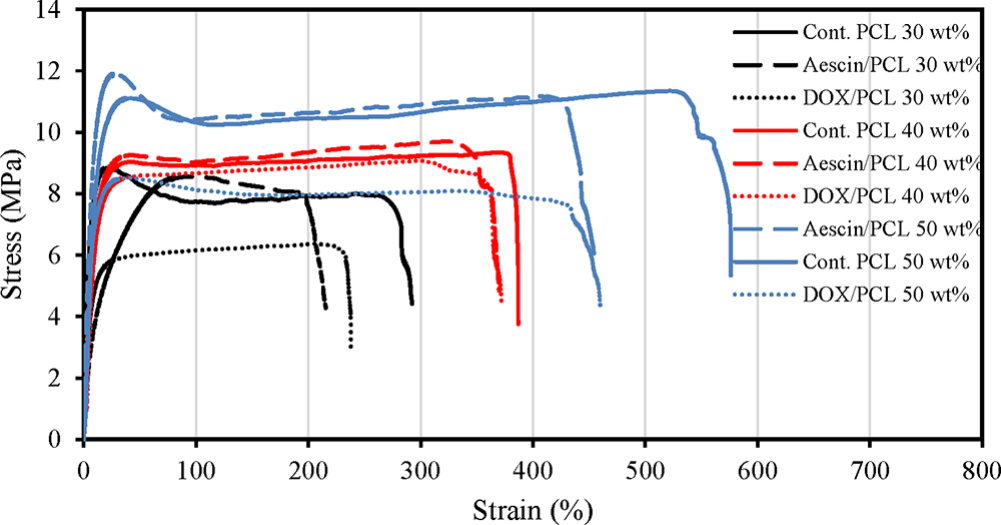
Mechanical properties of SMP films with a cross-linking thickness of 0.5 mm at room temperature. The stress–strain curves of different weight percents of 4b50PCL (30, 40, 50 wt %) with and without drugs (Aescin and doxorubicin (Dox)).

The thermal properties of the SMPUSs were assessed using DSC analysis. The degree of crystallinity (χ_c_) of the SMPUSs loaded with drugs slightly decreased compared to the SMPUSs without drugs at the same 4b50PCL concentrations. As depicted in Figure 2, the shape transition temperatures (*T*_m_) of the cross-linked 4b50PCL SMPUSs with a thickness of 0.5 mm prepared from 30, 40, and 50 wt % polymer solutions without drugs were 55.9, 52.3, and 52.2 °C, respectively, while the χ_c_ values were 41.3, 36.5, and 35.9%, respectively. The *T*_m_ of 4b50PCL and 4b50PCL macromonomers was analyzed, showing temperatures of 55.5 and 54.9 °C, respectively. Interestingly, the *T*_m_ of all of the drug-containing SMPUSs ranged from 47 to 53 °C, with the *T*_c_ ranging from 27.4 to 31.8 °C, while the χ_c_ ranged from 25.3 to 32.7%. These data indicate the successful fabrication of cross-linked 4b50PCL SMPUSs with drugs within the required temperature ranges, particularly with shape transition temperatures below 55 °C, suitable for the used temperature (50–55 °C) of solutions during the urethral stents’ surgeries.^35,36^ The *T*_c_ of all drug-loaded SMPUSs is around 30 ± 2 °C, allowing easy shape fixation after cooling down the stents.

**Figure 2:**
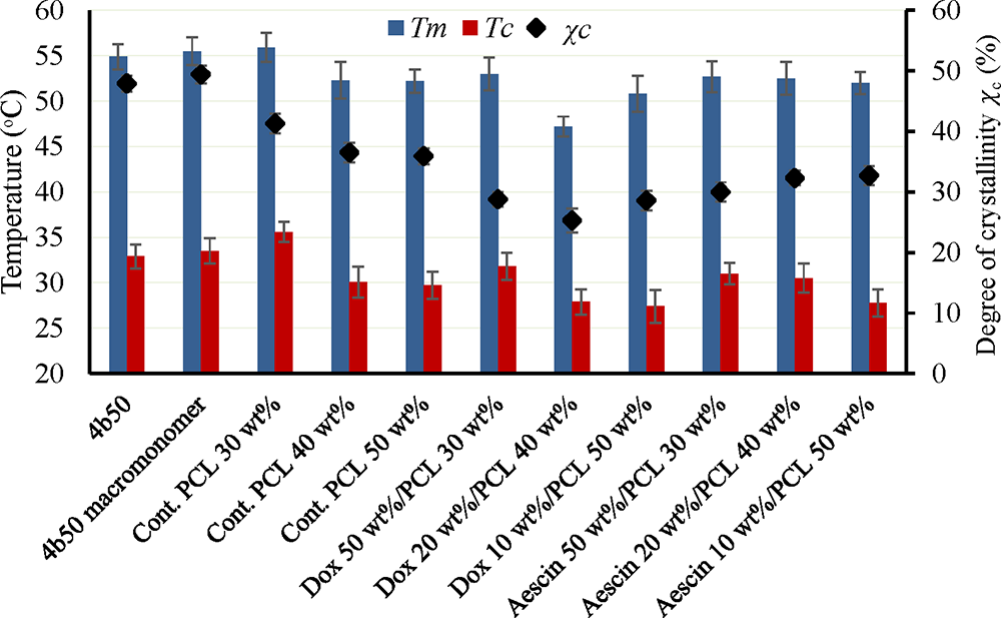
Thermal properties of the SMPUS. The samples were analyzed using DSC. Melting temperature (*T*_m_), crystallization temperature (*T*_c_), and degree of crystallinity (χ_c_) of premacromonomerized polymer (4b50), 4b50 macromonomer, and the cross-linked product of different 4b50PCL concentrations (30, 40, 50 wt %), with and without drugs (Aescin and doxorubicin (Dox)) at a thickness of 0.5 mm are presented. Data were obtained from three independent replicates (*n* = 3). Data are presented as the mean and SD.

### 3.2 Drug Release Profile of the Fabricated SMPUSs

In this study, the drug release profiles of the fabricated SMPUSs were evaluated. Both drugs Doxorubicin (Dox) and Aescin were tested with different concentrations of 10, 20, and 50 wt %. Figure 3a shows the obtained calibration curve of the fluorescence intensity of Dox dissolved in a 1:1 mixture of PBS and DMF, which was used for the calculation of the released concentrations of Dox. The release of Dox from each SMPUS was gradually increased, showing sustained release throughout the evaluation period (60 days), as shown in Figure 3b. A higher initial concentration of loaded Dox resulted in a higher percentage of drug release. After 60 days, the Dox release efficiencies were 13.1, 35.2, and 49.5% for the SMPUSs with 10, 20, and 50 wt %, respectively.

**Figure 3:**
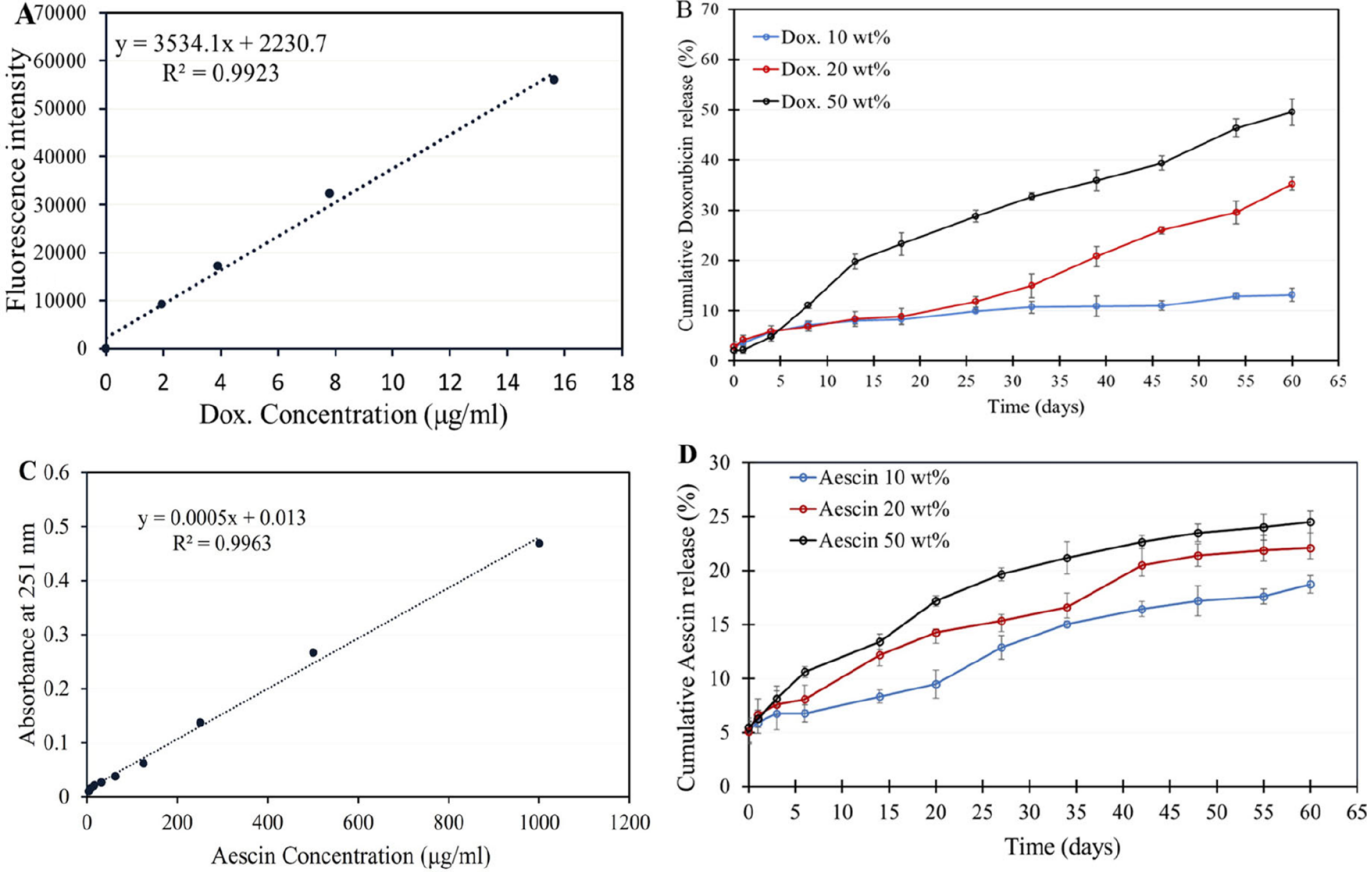
Collection of the cumulative release percentage of drugs from drug-loaded SMPUSs for 60 days. (A) Calibration curve of the fluorescence intensity of doxorubicin (Dox) dissolved in a 1:1 mixture of PBS and DMF; doxorubicin fluorescence signals were measured at a wavelength of 480 nm for excitation and 490 nm for emission using a plate reader. (B) Cumulative release percent of Dox (10, 20, 50 wt %) from SMPUS. (C) Calibration curve of the absorbance of Aescin dissolved in PBS; Aescin absorbance was measured at 251 nm by a spectrophotometer. (D) Cumulative release percent of Aescin (10, 20, 50 wt %) from SMPUS. The drug release assay was performed 3 times each at 37 °C for 60 days. Data are presented as mean and SD.

Aescin release profile was calculated based on the obtained calibration curve of the absorbance of Aescin dissolved in PBS (Figure 3c). The release behavior commenced with an initial burst of approximately 5%, followed by sustained release that increased with the loaded amount of the drug, as shown in Figure 3d. After 60 days, the release efficiencies were 18.7, 22.1, and 24.5% for the SMPUSs with 10, 20, and 50 wt %, respectively.

The actual loaded concentrations of drugs in SMPUSs were calculated by subtracting the released amounts in the wash solutions from the initial feed amounts. The washing process aimed to remove the nonincorporated molecules. Approximately 3–5% of the initial feed amounts of drugs could be washed out by the quick washing process that we applied.

### 3.3 SMPUS Efficiency in Mechanical Treatment of PUO

To confirm the mechanical strength of the fabricated SMPUS in maintaining the patency of the obstructed prostatic urethra after shape recovery, we utilized a porcine model of PUO. In comparison to the normal prostatic urethra, partial and severe urethral obstructions were established by applying external pressure using the rubber band (Figure 4a). Subsequently, SMPUSs composed of 40 wt % 4b50PCL were inserted in their temporary folded shape (Figure 4b,c) into the obstructed urethras as an average percentage of the fabricated SMPUSs. Cross sections at the level of the internal urethral opening were grossly checked before and after insertion of the SMPUSs as shown in Figure 4d. The permanent shape was restored by flushing with hot water (50–55 °C) and fixed by cooling, allowing the SMPUSs to keep the urethral lumen open in both partial and severe PUO models.

**Figure 4:**
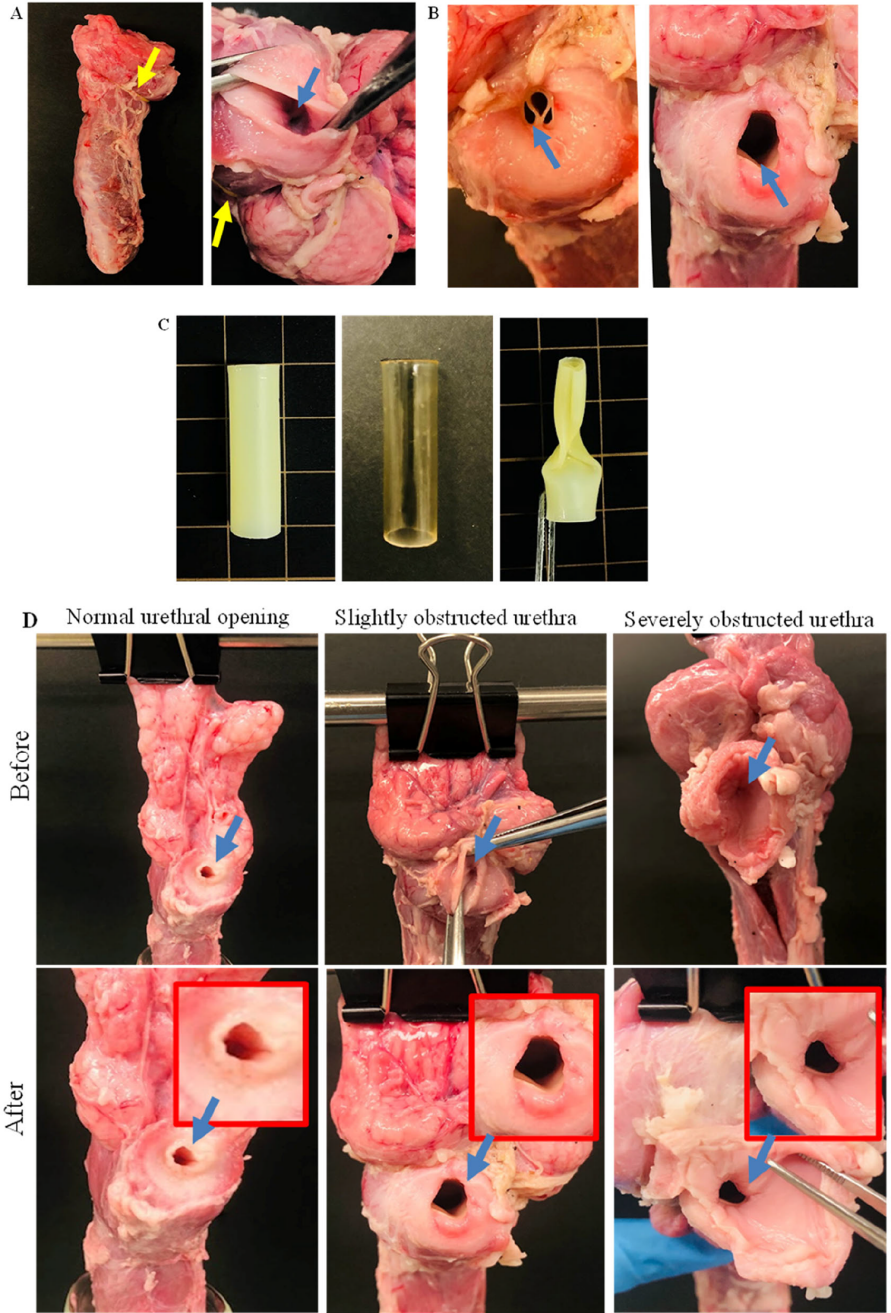
Efficiency of SMPUS to maintain the patency of the obstructed prostatic urethra after shape recovery. (A) Images of using the rubber band to establish the obstructed prostatic urethra; yellow arrows show the place of the band. (B) SMPUS before (folded) and after (opened) the shape recovery inside the in vitro-mimicked obstructed prostatic urethra. Shape was restored by flushing with hot water (50–55 °C). (C) Images of the permanent (off-white), temporary (transparent), and folded states of the used SMPUS. (D) Images of cross sections of the internal urethral opening before and after insertion of the SMPUSs into the obstructed prostatic urethra. Blue arrows show the prostatic urethra at the opening level.

Additionally, the water-flow efficiency of SMPUSs in the in vitro-mimicked model was checked by measuring the volumes of water that flowed before and after inserting the SMPUSs into the obstructed prostatic urethra within 30 s as shown in Figure 5a. In the model representing the normal urethra, 61 mL of water could flow smoothly, whereas in the case of slight and severe PUO, only 40 and 19 mL of water could flow, respectively. Following the inserting of the SMPUSs, the volumes of the water that flowed increased to 54 and 52.7 mL in the cases of slight and severe PUO, respectively, as shown in Figure 5b. The results highlight the potential of the fabricated SMPUSs to maintain the patency of the obstructed urethra, facilitating smooth urine flow.

**Figure 5:**
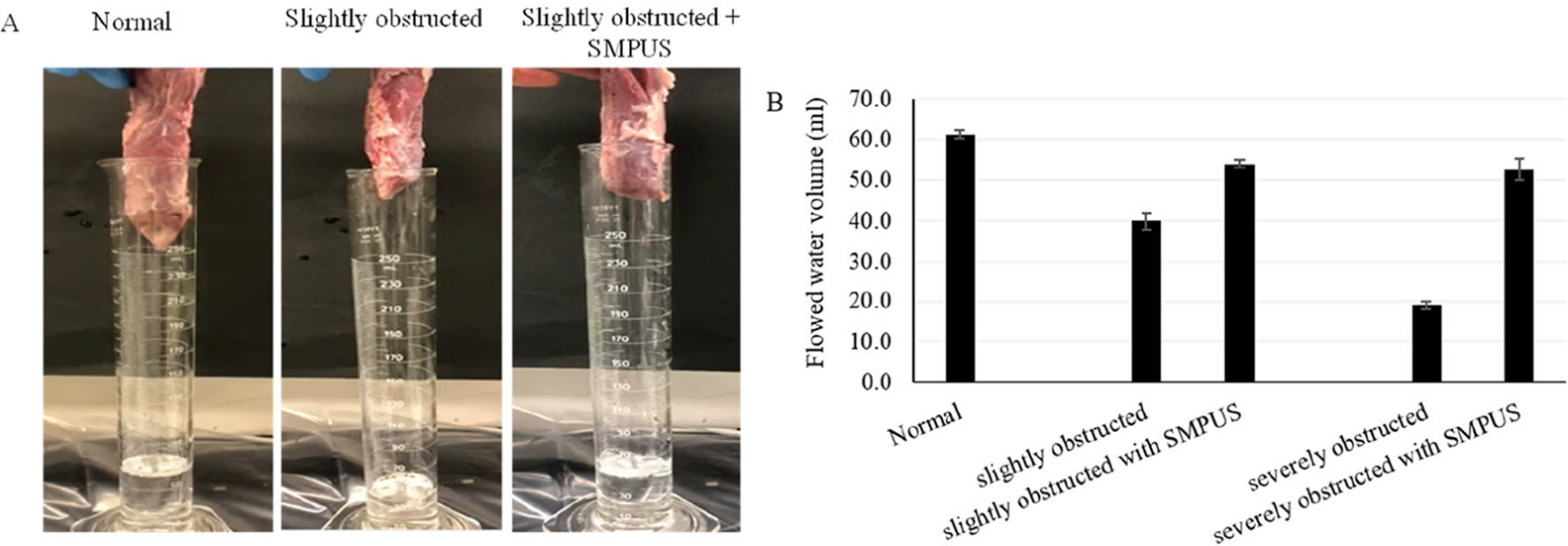
Water-flow efficiency of SMPUSs in in vitro-mimicked obstructed prostatic urethra. (A) Images of the water-flow mimicking process using normal and in vitro-mimicked obstructed porcine organs before and after the SMPUS insertion. (B) Volumes of water that flowed before and after inserting the SMPUSs (4b50 PCL 40 wt %) into the obstructed prostatic urethra within 30 s, presented as a mean of three repeats and the SD.

## 4 Discussion

In this study, we developed a drug-loaded SMPUS to integrate both mechanical and chemical/herbal therapies for treating PUO. As illustrated in Figure 6, the concept involves leveraging the shape-memory properties of the fabricated SMPUS. When the SMPUS is heated to its *T*_m_ or beyond, crystalline phases are melted, transitioning the phase into the amorphous state. At this stage, a bending motion along the SMPUS’s longitudinal axis is applied to temporarily deform the SMPUS into a folded shape with a very thin diameter. This is subsequently followed by a cooling down to reform the crystalline phases and fix the folded temporary shape for easy insertion into the obstructed urethra through the external urethral orifice. After insertion into the prostatic urethra, the SMPUS is heated again by flushing with hot water (*T*_m_) to recover its permanent tube-like shape. Then it is cooled by flushing cold water (*T*_c_) to enable the tube-like shape fixity, keeping the prostatic urethral lumen open. The SMPUSs are fabricated using biodegradable shape-memory PCL and loaded with drugs for localized therapeutic effects. PCL, an FDA-approved synthetic polymer, is widely used in medical applications owing to its biocompatibility and biodegradability,^37,38^ with proven nontoxic effects in various laboratory animals' models.^39^

**Figure 6:**
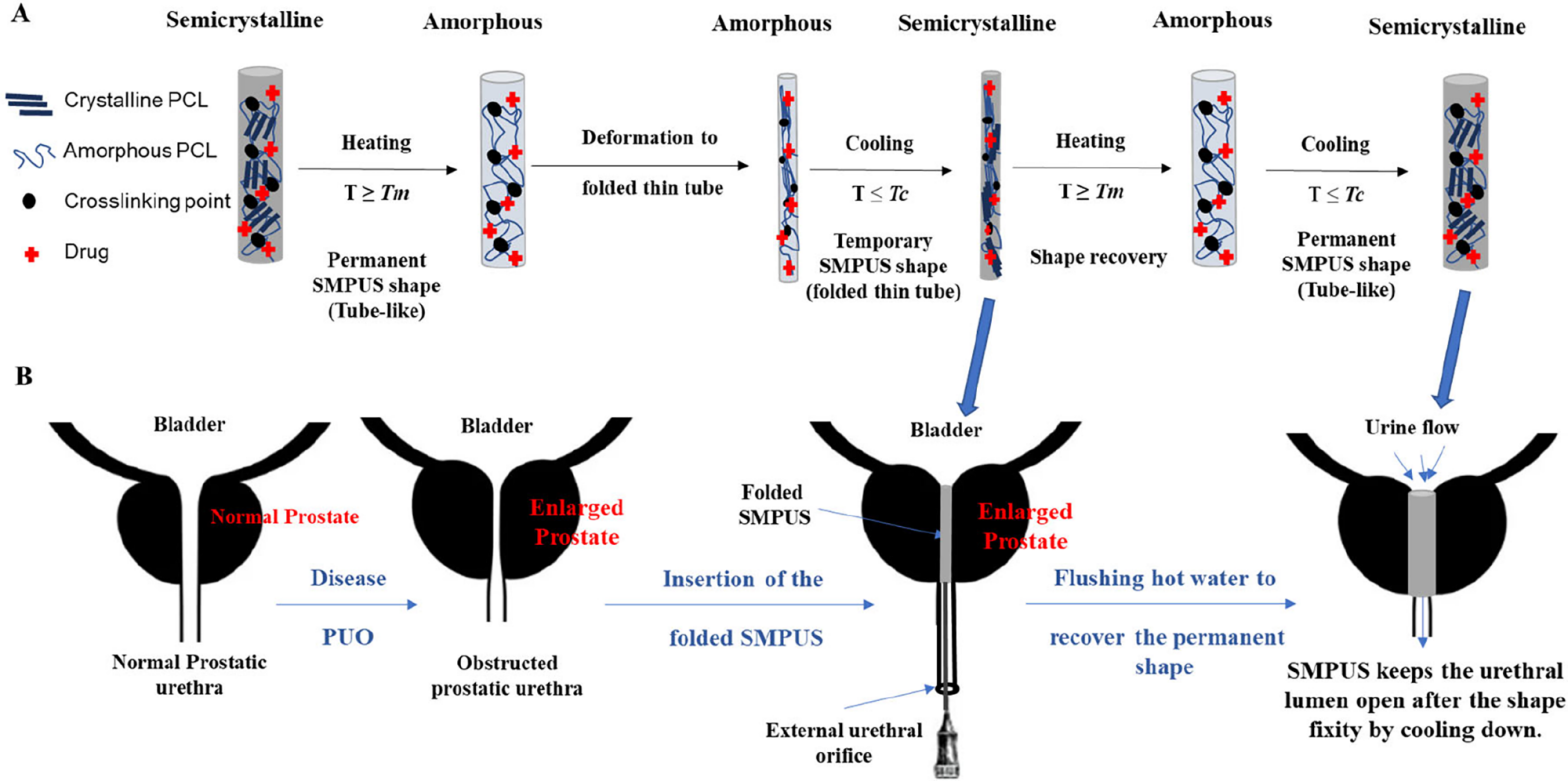
Schematic illustration of (A) the mechanism of the shape-memory property of SMPUS, and (B) the concept of using SMPUS to open the obstructed prostatic urethra.

The newly developed SMPUSs were fabricated by cross-linking the synthesized 4b50PCL macromonomer with polymer concentrations of 30, 40, and 50 and 2 wt % of the BPO as a thermal initiator. This process was conducted in the presence of the selected drug after all of the drug was dissolved in the DMF solvent. The resulting cross-linked 4b50PCL exhibits the shape-memory properties required for the current application of SMPUS and shows advantages over the currently used stents in terms of biodegradability and stimuli-response (temperature in this study).

To ensure the successful application of SMPUS, both mechanical and thermal properties must be considered. Critical factors for the medical application of SMPUS in treating PUO conditions are mechanical strength and shape transition temperature (*T*_m_). Our results indicate that the *T*_m_ and χ_c_ of the SMPUS were slightly reduced by increasing the polymer concentration, with no significant differences observed in the drug-loaded SMPUSs with the same polymer concentration. These findings are attributed to the denser environment of the mixture at higher polymer concentrations, resulting in increased cross-linking and reduced crystallinity, leading to a decrease in the *T*_m_ and χ_c_.^28,30^ Our aim was to develop the SMPUS with *T*_m_ below 55 °C for easy application in PUO treatment, achieved by simple flushing with hot water within the temperature range 50–55 °C, which was used in urethral stents’ surgeries.^35,36^ Successfully, as shown in Figure 2, the *T*_m_ of all of the drug-containing SMPUSs ranged from 47 to 53 °C, allowing for straightforward medical application. In terms of the *T*_c_ of the drug-loaded SMPUSs, it ranged from 27.4 to 31.8 °C, easily attainable by flushing water during medical application to ensure the crystallinity and shape fixity of the SMPUS.

The final dimensions of the fabricated SMPUS were 10 mm in diameter, 30 mm in length, and 0.5 mm in thickness, aligning with the recommended dimensions of the available prostatic stents ranging from 7 to 11 mm in diameter, 20–50 mm in length, and 0.4–1 mm in thickness,^40,41^ depending on the individual variations among patients. Thickness is a crucial factor in the cross-linking process, and it was selected to be the thinnest (0.5 mm) based on our previously published data, indicating an increase in the *T*_m_ with thicker films due to heat penetration delay.^30^ Additionally, it was noted that after the cross-linking, the SMPUSs had a slightly lower thickness than during the cross-linking process in the Teflon mold.

Moreover, the mechanical strength of SMPUS to restore its permanent shape is crucial for reopening the obstructed prostatic urethral lumen under the external pressure of the enlarged prostate. As illustrated in Figure 6, the concept of shape-memory properties involves heating the SMPUS to the *T*_m_ or above, inducing entropic change and increased chain mobility in the cross-linked network. After deforming the SMPUS into the folded temporary shape and cooling it down to the *T*_c_ or below, the temporary shape is fixed, storing the acquired unstable energy. This stored energy is released upon reheating the SMPUS, facilitating the recovery of the permanent tube-like shape. To assess the strength of the fabricated SMPUSs, films were prepared from the SMPUS mixtures with varying polymer concentrations (30, 40, and 50 wt %) and evaluated using a tensile tester. The stress–strain curves (Figure 1) were obtained by subjecting the cross-linked films to stretching until overload. The data shows that the elongation (strain %) required to deform the material increased with a higher polymer concentration, ranging from 280% in 30 wt % polymer to 550% in 50 wt % polymer. Similarly, the force required to deform the material increased with a higher polymer concentration, rising from ∼9 MPa in 30 and 40 wt % up to ∼12 MPa in 50 wt %, confirming that heightened cross-linking density reduces the crystallinity at higher polymer concentrations. Regarding the drug-loaded SMPUSs, the required force (stress) and elongation % (strain) were slightly reduced compared to the nonloaded SMPUSs with the same polymer concentration. However, these reductions are within the acceptable ranges for the current application. The obtained stress and strain results confirm the mechanical capability of the SMPUSs to recover their shape in the prostatic urethral lumen against the induced external pressure by the enlarged prostate. The 50 wt % polymer exhibits optimal strength, but it cannot accommodate higher concentrations of drugs due to the solvent’s solubility saturation. On the other hand, the 30 wt % polymer could accommodate the highest drug concentrations but exhibited the lowest strength to achieve the restoring of the permanent tube-like stent shape inside the urethra under the external pressure of the enlarged prostate. This is attributed to the reduction of the actual thickness compared to the intended one (0.5 mm) during the cross-linking process in the Teflon mold. The thickness of 30 wt % 4b50PCL films decreased to approximately 0.22 mm, which subsequently reduced their strength compared to the 40 and 50 wt % 4b50 PCL films with actual thicknesses of approximately 0.32 and 0.38 mm, respectively. However, the SMPUS fabricated with 30 wt % 4b50PCL can be employed to maintain the patency of the obstructed prostatic urethra through a simple modification of the application process. It could be inserted a little more inside the urinary bladder and allowed to restore its permanent shape by flushing with hot water, followed by cold water flushing to allow its crystallization before pulling it back by a forceps to the level of the prostatic urethra to keep it open. Therefore, the selection of both polymer and drug concentrations will depend on the severity of the PUO, the size of the enlarged prostate, and the progress of its external pressure. To investigate the efficiency of SMPUS in keeping the prostatic urethra open, an in vitro model of male porcine organs mimicking PUO conditions (slight and severe) through external prostate pressure was used, as shown in Figure 4. Cross sections at the level of the internal urethral opening were grossly examined before and after the SMPUS insertion, and the results were compared to the normal model. Various SMPUSs with different polymer concentrations (30, 40, and 50 wt %) were tested, and the results of the SMPUS with 40 wt % as an average concentration are shown in Figure 4. The findings demonstrate the recovery from the temporary folded shape of SMPUS to its permanent tube-like shape in both slight and severe PUO models. Notably, the external pressure in the severe PUO model induced a slight compromise in the circular shape of the tube lumen, as shown in Figure 4d, without impacting the functional efficiency to allow urine flow. To confirm the urine flow efficiency, a controlled peristaltic pump was used to mimic the process, maintaining relatively constant pressure within 30 s (Figure 5a). SMPUS retrieved the volumes of the water that flowed up to 54.0 and 52.7 mL in slight and severe PUO, respectively, compared to 61.3 mL in the case of the normal model (Figure 5b), showcasing its potential efficiency in mechanically treating the PUO as a primary target.

The efficiency of drug-loaded SMPUS in treating the condition that leads to PUO through the localized chemical effect of the selected drugs is the second objective of this study. Two drugs were selected for their relevance to the primary causes of prostate enlargement associated with PUO. The first is Dox, a commonly used chemotherapeutic agent with a proven strong antitumor effect in the case of PCa.^42^ The second drug is Aescin, a herbal drug commonly used for BPH treatment. Aescin is extracted from *Aesculus hippocastanum* (horse chestnut) seeds and consists of triterpene saponin glycosides, which contribute to its pharmacological effects.^43,44^ Aescin has been reported for its potential protective role against BPH via its anti-inflammatory and antiproliferative effects in laboratory animals.^45,46^ Despite being a generally safe drug, Aescin is associated with side effects such as weakness, depression, incoordination, and GIT disturbance.^47^ Therefore, in this study, we explore its potential for local release to avoid systemic side effects in future applications. To investigate the drug release profile of both drugs, SMPUSs were loaded with different concentrations of drugs based on the maximum amount soluble in the DMF with a specific concentration of 4b50 PCL. Ultimately, SMPUSs were fabricated with 50, 20, and 10 wt % of drugs against 30, 40, and 50 wt % of the polymer, respectively. To achieve the desired chemical therapy, the loaded drug concentration must be increased, requiring a reduction in the polymer concentration responsible for the mechanical therapy. Consequently, selecting the optimal conditions of the SMPUS depends on the progression of the disease and the degree of PUO on a case-by-case basis. The current findings (Figure 3b) depict the gradual release of Dox over 60 days at 37 °C, with an increased percentage of released drug corresponding to the higher concentration loaded into the SMPUS. A similar trend is observed in the Aescin release profile (Figure 3d), which exhibits an initial burst release of approximately 5%, followed by sustained release. After 60 days, SMPUSs loaded with 50 wt % of both Dox and Aescin drugs achieved release percentages of 49.5% and 24.5%, respectively. This variance in the drug release behavior is primarily attributed to the hydrophilicity and solubility of drugs, influencing their incorporation into the PCL-based SMPUSs and subsequent release in PBS. Dox has been extensively studied for its solubility in the organic solvent DMF, allowing its incorporation into the SMPUS mixture through ultrasonication as demonstrated in our previous study.^28^ Its release in PBS from PCL nanofiber mesh has been reported previously^48^ and is corroborated in this study as well. Concerning Aescin, its chemical structure comprises a lipophilic sapogenin aglycon and water-soluble glycan chain, indicating its amphiphilic nature that enhances its bioactivity.^49,50^ This clarifies the successful incorporation of Aescin into the fabricated SMPUSs and its subsequent release behavior in the PBS solution. The drug release profiles of both compounds in vitro demonstrate the possibility of localized drug release in the tissues and the potential application of the newly developed SMPUSs in local drug delivery after further investigation of the drug release in an in vivo model. Several factors could be modulated to achieve the desirable effects concerning required doses and administration times for further medical applications.

## 5 Conclusions

In this study, we successfully developed a drug-loaded SMPUS designed to address PUO. The SMPUS demonstrates mechanical efficacy through its shape-memory properties that ensure the patency of the urethral lumen by restoring its tube-like stent shape. Moreover, the in vitro drug release investigation shows the capability of SMPUS to serve as a versatile drug delivery platform, after further in vivo investigation. This capability holds promise for achieving targeted chemical or herbal therapeutic effects against the primary diseases associated with PUO while mitigating the systemic side effects associated with conventional treatments. The adaptability of the developed SMPUS allows for future in vivo and clinical trials in medical applications.

## Data Availability

The authors declare that all data supporting the findings of this study are available within the paper. Additional data related to this paper may be requested from the authors.

## □

Department of Materials Science and Engineering, School of Materials and Chemical Technology, Institute of Science Tokyo, Yokohama, Kanagawa 226-8503, Japan

## References

[1] Coakley F. V., Hricak H. (2000). Radiologic anatomy of the prostate gland: A clinical
approach. Radiol Clin North Am..

[2] Ferlay J., Soerjomataram I., Dikshit R. (2015). Cancer incidence and
mortality worldwide: Sources, methods and major patterns in GLOBOCAN
2012. Int. J. Cancer..

[3] Awedew A. F., Han H., Abbasi B. (2022). The global, regional, and national burden of
benign prostatic hyperplasia in 204 countries and territories from
2000 to 2019: a systematic analysis for the Global Burden of Disease
Study 2019. Lancet Healthy Longevity.

[4] Lorenzo G., Hughes T. J. R., Dominguez-Frojan P. (2019). Computer simulations
suggest that prostate enlargement due to benign prostatic hyperplasia
mechanically impedes prostate cancer growth. Proc. Natl. Acad. Sci. U. S. A..

[5] Komninos C., Mitsogiannis I. (2014). Obstruction-induced
alterations within the urinary
bladder and their role in the pathophysiology of lower urinary tract
symptomatology. Can. Urol. Assoc. J..

[6] Nordling J. (1994). Definition
of prostatic urethral obstruction. Urol. Res..

[7] Peyton C. C., Badlani G. H. (2015). The management of
prostatic obstruction with urethral
stents. Can. J. Urol..

[8] Rassweiler J., Teber D., Kuntz R. (2006). Complications of transurethral
resection of the prostate (TURP)--incidence, management, and prevention. Eur. Urol..

[9] Sagen E., Nelzén O., Peeker R. (2020). Transurethral resection
of the prostate:
fate of the non-responders. Scand J. Urol..

[10] Fabian K. M. (1980). Der Intraprostatische
“Partielle Katheter” (Urologische Spirale) [The intra-prostatic
“partial catheter” (urological spiral) (author’s
transl)]. Urologe A.

[11] Geavlete, P ; Niţă, G ; Mulţescu, R , Endoscopic Diagnosis and Treatment in Prostate Pathology; Elsevier Academic Press, 2016; Chapter 11, Prostatic Stents; pp 161–170.

[12] Sountoulides P., Karatzas A., Gravas S. (2019). Current and
emerging mechanical minimally
invasive therapies for benign prostatic obstruction. Ther. Adv. Urol..

[13] Uchikoba T., Horiuchi K., Satoh M. (2005). Urethral
stent (Angiomed-Memotherm)
implantation in high-risk patients with urinary retention. Hinyokika Kiyo..

[14] Staios D., Shergill I., Thwaini A. (2007). The MemokathTM stent. Expert Review of Medical Devices..

[15] Tomschi W., Lüftenegger W. (1990). The urological
spiral. A real alternative to the indwelling
catheter? Experience in 23 patients. Wien. Klin.
Wochenschr..

[16] van
Dijk M. M., Mochtar C. A., Wijkstra H. (2006). The bell-shaped
nitinol prostatic stent in the treatment of lower urinary tract symptoms:
experience in 108 patients. Eur. Urol..

[17] Grimsley S. J., Khan M. H., Lennox E. (2007). Experience with the
spanner prostatic stent in patients unfit for surgery: an observational
study. J. Endourol..

[18] Kotsar A., Isotalo T., Juuti H. (2009). Biodegradable braided
poly­(lactic-co-glycolic acid) urethral stent combined with dutasteride
in the treatment of acute urinary retention due to benign prostatic
enlargement: a pilot study. BJU Int..

[19] Papatsoris A. G., Junaid I., Zachou A. (2011). New developments in
the use of prostatic stents. Open Access J.
Urol..

[20] Miernik A., Gratzke C. (2020). Current Treatment for Benign Prostatic Hyperplasia. Dtsch Arztebl Int..

[21] Nevedomskaya E., Baumgart S. J., Haendler B. (2018). Recent Advances
in Prostate Cancer
Treatment and Drug Discovery. Int. J. Mol. Sci..

[22] Ebara M., Uto K., Idota N. (2012). Shape-memory surface with dynamically tunable
nano-geometry activated by body heat. Adv. Mater..

[23] Shou Q., Uto K., Iwanaga M. (2014). Near-infrared light-responsive shape-memory
poly (ε-caprolactone) films that actuate in physiological temperature
range. Polym. J..

[24] Muroya T., Yamamoto K., Aoyagi T. (2009). Degradation
of cross-linked aliphatic
polyester composed of poly (ε-caprolactone-co-d, l-lactide)
depending on the thermal properties. Polymer
degradation and stability.

[25] Uto K., Yamamoto K., Hirase S. (2006). Temperature-responsive
cross-linked poly (ε-caprolactone) membrane that functions near
body temperature. Journal of controlled release..

[26] Zhang Y. F., Zhang N., Hingorani H. (2019). Fast-response, stiffness-tunable
soft actuator by hybrid multimaterial 3D printing. Adv. Funct. Mater..

[27] Zou M., Li S., Hu X. (2021). Progresses in tensile, torsional, and multifunctional
soft actuators. Adv. Funct. Mater..

[28] Ouchi S., Niiyama E., Sugo K. (2021). Shape-memory
balloon
offering simultaneous thermo/chemotherapies to improve anti-osteosarcoma
efficacy. Biomaterials Science..

[29] Baer G. M., Small W., Wilson T. S. (2007). Fabrication and in vitro
deployment of a laser-activated shape memory polymer vascular stent. Biomed. Eng. Online.

[30] Fulati A., Uto K., Iwanaga M. (2022). Smart Shape-Memory Polymeric String for
the Contraction of Blood Vessels in Fetal Surgery of Sacrococcygeal
Teratoma. Adv. Healthc. Mater..

[31] Niiyama E., Uto K., Lee C. M. (2019). Hyperthermia
Nanofiber Platform Synergized
by Sustained Release of Paclitaxel to Improve Antitumor Efficiency. Adv. Healthc. Mater..

[32] Oe E., Fujisawa N., Chen L. (2023). Locally implantable
nanofibre meshes by sustained release of Temozolomide for combined
thermo-chemotherapy to treat glioblastoma. New
J. Chem..

[33] Han C., Ran X., Su X. (2007). Effect of peroxide crosslinking on thermal
and mechanical properties of poly (ε-caprolactone). Polymer international..

[34] Chandrasekaran A. R., Jia C. Y., Theng C. S. (2011). Invitro studies and
evaluation of metformin marketed tablets-Malaysia. J. Appl. Pharm. Sci..

[35] Perry M. J., Roodhouse A. J., Gidlow A. B. (2002). Thermo-expandable intraprostatic
stents in bladder outlet obstruction: an 8-year study. BJU Int..

[36] Takahashi R., Kimata R., Hamasaki T. (2013). Memokath­(TM) urethral
stents induce incontinence in patients with urethral balloon catheters. J. Nippon Med. Sch..

[37] Choi Y. E., Battelli C., Watson J. (2014). Sublethal concentrations
of 17-AAG suppress homologous recombination DNA repair and enhance
sensitivity to carboplatin and olaparib in HR proficient ovarian cancer
cells. Oncotarget..

[38] Jirofti N., Mohebbi-Kalhori D., Masoumi R. (2022). Enhancing biocompatibility of PCL/PU
nano-structures to control the water wettability by NaOH hydrolysis
treatment for tissue engineering applications. Journal of Industrial Textiles..

[39] Jesus S., Bernardi N., da Silva J. (2020). Unravelling the Immunotoxicity
of Polycaprolactone Nanoparticles-Effects of Polymer Molecular Weight,
Hydrolysis, and Blends. Chem. Res. Toxicol..

[40] Mori K., Okamoto S., Akimoto M. (1995). Placement of the urethral stent made
of shape memory alloy in management of benign prostatic hypertrophy
for debilitated patients. J. Urol..

[41] Zhu Y., Yang K., Cheng R. (2017). The current status of
biodegradable stent to treat benign luminal disease. Materials Today..

[42] Kciuk M., Gielecińska A., Mujwar S. (2023). Doxorubicin-An Agent
with Multiple Mechanisms of Anticancer Activity. Cells..

[43] Gallelli L., Cione E., Wang T. (2021). Glucocorticoid-Like
Activity of Escin: A New Mechanism for an Old Drug. Drug Des Devel Ther..

[44] Idris S., Mishra A., Khushtar M. (2020). Phytochemical, ethanomedicinal and
pharmacological applications of escin from Aesculus hippocastanum
L. towards future medicine. J. Basic Clin Physiol
Pharmacol..

[45] Raafat M., Kamel A. A., Shehata A. H. (2022). Aescin protects against
experimental benign prostatic hyperplasia and preserves prostate histomorphology
in rats via suppression of inflammatory cytokines and cox-2. Pharmaceuticals..

[46] Tan S. M., Li F., Rajendran P. (2010). Identification of beta-escin as a novel
inhibitor of signal transducer and activator of transcription 3/Janus-activated
kinase 2 signaling pathway that suppresses proliferation and induces
apoptosis in human hepatocellular carcinoma cells. J. Pharmacol Exp Ther..

[47] Hess, H. M. Clinical Pharmacology During Pregnancy, 2nd ed.; Elsevier Academic Press, 2022; Chapter 21, Herbs and alternative remedies; pp 377–387.

[48] Chen L., Nabil A., Fujisawa N. (2023). A facile, flexible,
and multifunctional thermo-chemotherapy system for customized treatment
of drug-resistant breast cancer. J. Controlled
Release.

[49] Dias M. I., Albiston C., Anibarro-Ortega M. (2022). Sonoextraction of phenolic
compounds and saponins from Aesculus hippocastanum seed kernels: Modeling
and optimization. Industrial Crops and Products..

[50] Liao Y., Li Z., Zhou Q. (2021). Saponin surfactants used in drug delivery systems:
A new application for natural medicine components. International journal of pharmaceutics..

